# Pregnancy course and outcomes of patients with polymyositis and dermatomyositis (PM/DM) managed in a single center

**DOI:** 10.1097/MD.0000000000033462

**Published:** 2022-04-07

**Authors:** Rina Mino, Hiromi Shimada, Risa Wakiya, Shusaku Nakashima, Taichi Miyagi, Koichi Sugihara, Yusuke Ushio, Mao Mizusaki, Kanako Chujo, Tomohiro Kameda, Kenji Kanenishi, Norimitsu Kadowaki, Hiroaki Dobashi

**Affiliations:** a Department of Internal Medicine, Division of Hematology, Rheumatology and Respiratory Medicine, Faculty of Medicine, Kagawa University, Kagawa, Japan; b Department of Perinatology and Gynecology, Faculty of Medicine, Kagawa University, Kagawa, Japan.

**Keywords:** dermatomyositis, polymyositis, pregnancy, pregnancy outcomes

## Abstract

We aimed to determine the association between disease activity during pregnancy and pregnancy outcomes of women with polymyositis and dermatomyositis (PM/DM). Patients with PM/DM who were managed from pregnancy to delivery at Kagawa University Hospital from March 2006 to May 2021 were enrolled. Clinical data were retrospectively analyzed to evaluate the association between disease activity during pregnancy and pregnancy outcomes. Eight pregnancies in 5 women with PM/DM were analyzed. The mean age at conception was 28.3 ± 3.8 years, and mean disease duration was 6.3 ± 3.2 years. Four patients required an increased glucocorticoid dosage because of worsening disease activity (sustained elevation of creatine phosphokinase [CPK] concentration). Two patients who continuously received immunosuppressive drugs from conception to delivery showed no increase in disease activity and did not need increased glucocorticoid dosages. The pregnancy outcomes were 1 spontaneous abortion and 7 live births. The mean gestation length was 35.3 ± 5.2 weeks, and mean birthweight was 2297.7 ± 1041.4 g. Five adverse pregnancy outcomes (APOs) occurred (2 preterm births and 4 low birthweights); most of these cases had sustained elevation of CPK concentration and increased glucocorticoid dosages. No APOs occurred in the 2 patients who received continuous immunosuppressive medication. Continued use of pregnancy-compatible medications and control of disease activity with lower glucocorticoid dosages in pregnancies with PM/DM may be important to achieve good pregnancy outcomes.

## 1. Introduction

Dermatomyositis (DM) and polymyositis (PM) are rare autoimmune diseases characterized by proximal and symmetrical muscle weakness and muscle inflammation.^[1,2]^ The estimated female:male ratio of polymyositis and dermatomyositis (PM/DM) is 2:1, and most patients are affected after childbearing age; only about 14% of cases present during reproductive age.^[3]^ There are fewer case reports and studies of pregnancies complicated by PM/DM than of pregnancies complicated by systemic lupus erythematosus (SLE) or rheumatoid arthritis (RA). Previous studies have demonstrated that women with RA, SLE, and antiphospholipid syndrome have a greater risk of adverse pregnancy outcomes (APOs) such as spontaneous abortion, stillbirth, preterm birth, low birthweight, and preeclampsia than do women without rheumatic diseases.^[4–6]^ Furthermore, these APOs in women with RA or SLE are significantly associated with the disease activity or treatment agents, especially glucocorticoids.^[7,8]^ Regarding pregnancies complicated by PM/DM, small studies and case reports have revealed an increase in disease flares during pregnancy resulting in poor pregnancy outcomes, including preterm birth or fetal loss.^[9–13]^ In addition, some case reports have described PM/DM onset during pregnancy or in the postpartum period.^[14–16]^ However, these studies and case reports did not describe the changes in PM/DM disease activity parameters such as the creatine phosphokinase (CPK) level during pregnancy or the association between disease activity and pregnancy outcomes.

We aimed to determine the association between clinical course and APOs in 8 pregnancies of 5 patients with PM/DM who underwent continuous pregnancy management from preconception to delivery at our institution.

## 2. Methods

### 2.1. Patients and data collection

We analyzed the data of patients with rheumatic diseases who delivered in Kagawa University Hospital from March 2006 to May 2021. PM/DM was diagnosed according to the diagnostic criteria of the Japanese Ministry of Health, Labor and Welfare.^[17]^ Patients with PM/DM who were treated and delivered at Kagawa University Hospital were included in the data analysis. We investigated the patients’ clinical background characteristics (age at conception and disease duration [defined as the period from disease onset to conception]) and treatment agents administered before and during pregnancy (glucocorticoid use, mean glucocorticoid dosage, and immunosuppressant use). The change in the serum CPK levels was used to assess the disease activity of PM/DM. Worsening disease activity was defined as a sustained elevation of the CPK level compared with the pre-pregnancy CPK level. For the evaluation of pregnancy outcomes, preterm birth was defined as delivery before 37 gestational weeks. Low birthweight was defined as a birthweight of <2500 g. Small for gestational age was defined as a birthweight under the 10th percentile.

### 2.2. Statistical analysis

All data were retrospectively collected from the medical records. Values are presented as mean ± standard deviation for continuous variables and as numbers (percentage) for categorical variables.

## 3. Results

**Figure 1.**
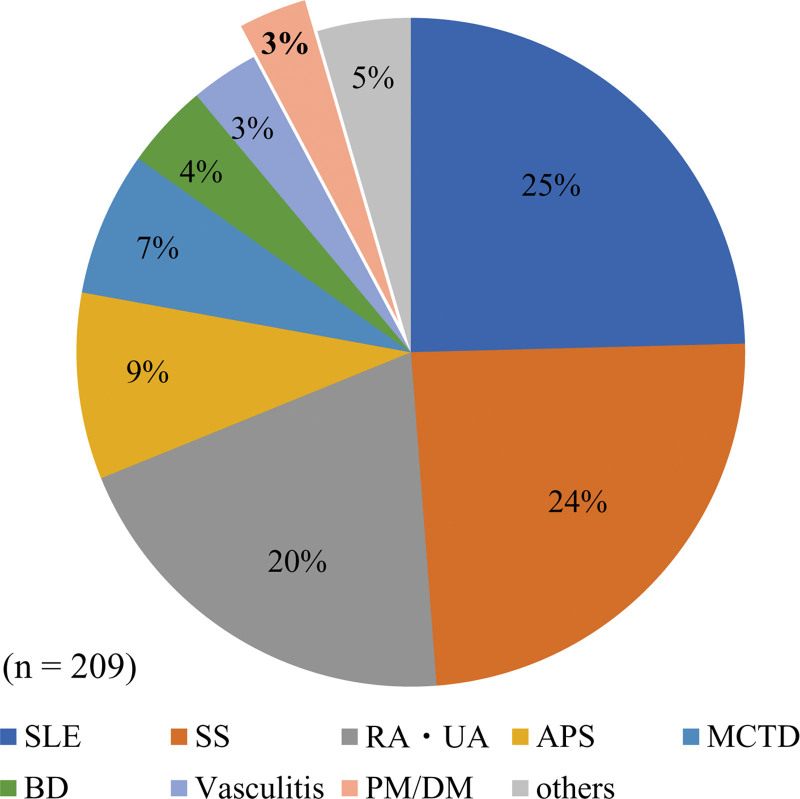
Pregnancies complicated by rheumatic disease in patients treated at our institution. APS = antiphospholipid syndrome, BD = Behçet’s disease, MCTD = mixed connective tissue disease, PM/DM = polymyositis/dermatomyositis, RA/UA = rheumatoid arthritis/undifferentiated arthritis, SLE = systemic lupus erythematosus, SS = Sjögren’s syndrome.

In total, 209 pregnancies complicated by rheumatic diseases were treated in our institution from March 2006 to May 2021. Among them, only 8 (3%) pregnancies occurred in 5 patients with PM/DM (Fig. 1).

Table 1 shows the 5 patients’ clinical characteristics and pre-pregnancy treatments. The patients in Cases 2 and 3 had multiple pregnancies (1 patient had 3 pregnancies [Cases 2a–c], and the other patient had 2 pregnancies [Cases 3a and b]). In these 8 cases, all patients received preconception counseling. However, only 5 pregnancies (Cases 1, 2c, 3a, 3b, and 5) were able to complete preconception care. The remaining 3 pregnancies did not complete preconception care. In other words, 5 pregnancies (Cases 1, 2c, 3a, 3b, and 5) were planned pregnancies and the rest was unplanned. The mean age at conception was 28.3 ± 3.8 years, and the mean disease duration was 6.3 ± 3.2 years. The autoantibody profiles of these patients are presented in Table 1. Glucocorticoids were administered before pregnancy in 7 cases (87.5%), and the mean glucocorticoid dosage was 5.8 ± 2.9 mg/day. Immunosuppressants were administered in 4 cases (50.0%). During the 12 months leading up to conception, serum CPK levels in our patients, which reflect the activity of myositis, were controlled within the normal range except 1 case (Case 2a). In this one case, the CPK level was persistently above the normal range and the patient required intensification of therapy comprising increased dosages of glucocorticoids. Therefore, this patient disease was considered active. The patient in Case 1 was diagnosed with and treated for SLE, and the disease activity of SLE in the 12 months before conception was inactive; however, she developed DM at 10 weeks.

**Table 1:** Clinical background characteristics and pre-pregnancy treatments of pregnant patients with polymyositis and dermatomyositis.

Case no.	Age at conception, yr	Disease duration, yr	Autoantibody profile	Disease status before conception	Interstitial lung disease	Pre-pregnancy treatments		
						Glucocorticoids use	Mean glucocorticoid dosage, mg/d	Immunosuppressants use
1	25	0	ANA (×640, homogenous, speckled), anti-dsDNA antibodies, anti-RNP antibodies, anti-Sm antibodies, anti-SS-A antibodies, anti-LAC antibodies	Pregnancy-induced	−	+	5	−
2a	25	1	ANA (×80, speckled, nucleolar, cytoplasmic), Anti-ARS antibodies (PL-7)	Active	+	+	10	+ (TAC)
2b	26	2		Inactive	+	+	7.5	+ (MTX)
2c	29	5		Inactive	+	+	5	+ (AZA)
3a	27	7	ANA: ND, anti-ARS antibodies	Inactive	+	+	3	−
3b	28	8		Inactive	+	+	8	−
4	28	6	Anti-SRP antibodies	Inactive	−	−	0	+ (AZA)
5	37	11	ANA (×1280, speckled, nucleolar)	Inactive	−	+	2	−
	28.3 ± 3.8	6.3 ± 3.2			5 (62.5)	7 (87.5)	5.8 ± 2.9	4 (50.0)

Data in the last row are presented as either mean ± standard deviation or n (%)

ANA = antinuclear antibody, ARS = aminoacyl tRNA synthetase, AZA = azathioprine, dsDNA = double-stranded DNA, LAC = lupus anticoagulant, MTX = methotrexate, ND = no data, RNP = ribonucleoprotein, SRP = signal recognition particle, TAC = tacrolimus.

Table 2 shows the changes in the serum CPK levels at the time of conception and during pregnancy under treatment with glucocorticoids and immunosuppressive drugs. The serum CPK level at the time of conception was within the reference range in all pregnancies except in Case 2a. However, 4 of 8 pregnancies (50.0%) had increased disease activity during pregnancy, as shown by a persistently elevated CPK level above the reference range. Of the 4 pregnancies with increased disease activity, the exacerbation occurred in the first trimester of pregnancy in 2 cases, the second trimester in 1, and the third trimester in 1. The mean glucocorticoid dosage during pregnancy was 10.9 ± 6.0 mg/day, and the maximum glucocorticoid dosage was 20 mg/day. In all 4 pregnancies with persistently elevated CPK levels, the patients required intensification of therapy comprising increased dosages of glucocorticoids during pregnancy. Only the patient in Case 1 received intravenous corticosteroid pulse therapy. She developed DM at 10 weeks and required intravenous corticosteroid pulse therapy because the disease activity was very high and difficult to control. In Case 3a, in which the CPK levels were not elevated during pregnancy, increased doses of glucocorticoids were required due to exacerbation of interstitial lung disease.

**Table 2:** CPK elevation and treatments during pregnancy in patients with polymyositis and dermatomyositis.

	CPK concentration (reference range: 41–153 U/L)				Treatment agents during pregnancy			
Case no.	CPK level before pregnancy, IU/mL	CPK elevation during pregnancy	Gestational wk at CPK elevation, IU/mL	Maximum CPK level	GC use	Mean GC dosage, mg/d	Increase in GC dosage	Immunosuppressant use
1	19	+	6	1488	+	20	+	−
2a	356	+	6	2895	+	15	+	−
2b	90	+	29	842	+	10.9	+	−
2c	57	−		52	+	5	−	+(AZA)
3a	65	−		75	+	12.5	+	−
3b	143	+	28	278	+	10.6	+	−
4	62	−		70	−	0	−	+(AZA)
5	61	−		50	+	2	−	−
	106.6 ± 106.7	4 (50.0)	17.3 ± 13.0	718.8 ± 1018.7	7 (87.5)	10.9 ± 6.0	5 (62.5)	2 (25.0)

Data in last row are presented as either mean ± standard deviation or n (%).

AZA = azathioprine, CPK = creatine phosphokinase, GC = glucocorticoid.

Two of 4 patients who were being treated with immunosuppressive drugs before pregnancy discontinued these drugs during pregnancy; this discontinuation was either because the drugs were not compatible with pregnancy or at the patient request. In the 2 pregnancies in which immunosuppressive drug therapy was continued during pregnancy, there was no worsening of disease activity.

The pregnancy outcomes are summarized in Table 3. Seven pregnancies ended in live births; however, the pregnancy in Case 2a ended in spontaneous abortion. The mean gestational age at delivery was 35.3 ± 5.2 weeks, and preterm birth occurred in 2 pregnancies. The mean newborn birthweight was 2297.7 ± 1041.4 g, and low birthweight occurred in 4 pregnancies (including 1 newborn who was small for gestational age). These APOs occurred in the patients with worsening disease activity, including persistently elevated CPK levels, and an increased glucocorticoid dosage. The pregnancies in which immunosuppressants were continued during pregnancy resulted in full-term births and normal birthweights.

**Table 3:** Pregnancy outcomes in patients with polymyositis and dermatomyositis.

Case no.	Outcome	Mode of delivery	Gestational age at delivery, wk	Birth weight of newborn, g	Apgar score at 1/5 min	Adverse pregnancy outcomes	NICU admission
1	Live birth	Cesarean section	26	590	2/7	Preterm birth, SGA, hypertensive disorders of pregnancy, eclampsia attack, HELLP syndrome	+
2a	Spontaneous abortion						
2b	Live birth	Cesarean section	30	1299	5/7	Preterm birth, LBW	+
2c	Live birth	Planned Cesarean section	38	2765	8/9	−	−
3a	Live birth	Transvaginal delivery	37	3290	8/9	−	−
3b	Live birth	Transvaginal delivery	37	2492	9/10	LBW	−
4	Live birth	Transvaginal delivery	39	3456	8/9	−	−
5	Live birth	Transvaginal delivery	40	2192	9/9	LBW	+
	7 live births 1 abortion		35.3 ± 5.2	2297.7 ± 1041.4			3 (42.9)

Data in last row are presented as either mean ± standard deviation or n (%)

HELLP = hemolysis, elevated liver enzymes, and low platelets, LBW = low birthweight, NICU = neonatal intensive care unit, SGA = small for gestational age.

## 4. Discussion

In this study, the clinical course and pregnancy outcomes of 8 pregnancies complicated by PM/DM were analyzed. In this case series, the patients in 4 of the 8 pregnancies required increased glucocorticoid dosages because of increased disease activity during pregnancy, and most of these cases ended in poor pregnancy outcomes. Previous studies of pregnancies complicated by PM/DM revealed an increase of disease activity during pregnancy that resulted in poor pregnancy outcomes, including preterm birth or fetal loss (Table 4).^[9–13]^ Most of these studies differ from our study in that they included patients without myositis or those who developed myositis after childbirth. In addition, these studies do not clearly state how they evaluated disease activity or defined disease exacerbation. In our study, worsening disease activity was defined as sustained elevation of the CPK level; 4 of 8 cases (50%) had worsening disease activity during pregnancy, which resulted in APOs.

**Table 4:** Disease activity and pregnancy outcomes of patients with myositis in the literature.

Authors	Reference no.	All cases of myositis, n	Myositis before pregnancy, n	Active cases during pregnancy n	Pregnancy loss, n (%)*	Live birth, n (%)*	Preterm birth, n (%)*	
Zhong et al	^[10]^	144	22	7	13 (59.1)	9 (40.1)	3 (13.6)	Higher risk of preterm birth or spontaneous abortion in women after PM/DM, especially in active state
Vancsa et al	^[13]^	186	14	6	6 (42.9)	6 (42.9)	2 (14.3)	Active disease resulting in 2 cases of prematurity, 4 cases of spontaneous abortion
Kolstad et al	^[11]^	853	ND	ND	ND	ND	ND	NIS dataset. IIMs are risk factors for hypertensive disorders and longer length of hospitalization
Nagy-Vincze et al	^[9]^	33	30	17 (56.7)	7 (23.3)	21 (70.0)	3 (10.0)	Disease activity before or during pregnancy is a risk factor for pregnancy complications
Che et al	^[12]^	801	68	ND	ND	ND	10 (14.7)	Higher risk for cesarean section, preterm birth, and low birthweight compared with non-IIM

IIM = idiopathic inflammatory myositis, ND = no data, NIS = National Inpatient Sample, PM/DM = polymyositis and dermatomyositis.

* Total number of patients with myositis before pregnancy.

APOs such as preterm labor, premature rupture of membranes, preeclampsia, and gestational diabetes are reportedly associated with glucocorticoid dosage and duration.^[18–22]^ Among the associations between glucocorticoid dosage and APOs, it was recently reported that glucocorticoid dosages above 20 mg/day in early and late pregnancy are associated with risk of preterm delivery.^[23]^ However, these studies did not clearly describe whether the changes in glucocorticoid dosages or disease activity were associated with APOs. In the present study, increased glucocorticoid dosages were required in 5 cases because of increased disease activity during pregnancy; this resulted in poor pregnancy outcomes in most of these cases. Our previous study showed that glucocorticoid treatment at a dosage of <20 mg/day is associated with preterm birth. We reported that in pregnancies complicated with SLE, the cutoff dosage of glucocorticoids affecting preterm birth is as low as 10 mg/day.^[24]^ In this study, preterm birth occurred in 2 pregnancies with glucocorticoid treatment at a mean dosage of <20 mg/day. These results suggest that even a glucocorticoid dosage of below 20 mg/day is associated with preterm birth.

In the present study, 3 of 4 pregnancies (including the 2 pregnancies in which azathioprine was continued during pregnancy) did not have worsened disease activity and resulted in no APOs associated with disease activity. Although the small sample size of our study makes it difficult to determine whether disease activity or glucocorticoid use was more closely associated with these APOs, the disease activity of patients with PM/DM should be managed with an appropriately lower glucocorticoid dosage during pregnancy. In Cases 2c and 4, we referred to the 2020 American College of Rheumatology guideline for the management of reproductive health in rheumatic and musculoskeletal diseases^[25]^ and decided to continue azathioprine because of its compatibility with pregnancy. As a result, the disease activity during pregnancy was not exacerbated and was controlled with a low dose of glucocorticoids. Therefore, the present findings suggest that continuing pregnancy-compatible immunosuppressants such as azathioprine may control the disease activity with a lower glucocorticoid dosage and lead to favorable pregnancy outcomes.

In conclusion, pregnancies complicated by PM/DM tended to show elevated disease activity and to require a higher glucocorticoid dosage, leading to poorer pregnancy outcomes. We suggest that continuing pregnancy-compatible immunosuppressants may control the disease activity with a lower glucocorticoid dosage and lead to favorable pregnancy outcomes.

## Acknowledgments

We thank Angela Morben, DVM, ELS, and Kelly Zammit, BVSc, from Edanz (https://jp.edanz.com/ac), for editing a draft of this manuscript.

## Author contributions

**Conceptualization:** Rina Mino, Hiromi Shimada, Hiroaki Dobashi.

**Data curation:** Hiromi Shimada.

**Formal analysis:** Rina Mino, Hiromi Shimada.

**Investigation:** Rina Mino, Hiromi Shimada.

**Supervision:** Rina Mino, Hiroaki Dobashi.

**Validation:** Rina Mino, Hiromi Shimada, Hiroaki Dobashi.

**Visualization:** Rina Mino, Hiromi Shimada.

**Writing – original draft:** Rina Mino, Hiromi Shimada.

**Writing – review & editing:** Rina Mino, Hiromi Shimada, Risa Wakiya, Shusaku Nakashima, Taichi Miyagi, Koichi Sugihara, Yusuke Ushio, Mao Mizusaki, Kanako Chujo, Tomohiro Kameda, Kenji Kanenishi, Norimitsu Kadowaki, Hiroaki Dobashi.
